# Metals and Alloys

**Published:** 1863-04

**Authors:** B. Wood

**Affiliations:** Albany, N. Y.


### DENTAL REGISTER OF THE WEST.

VOL. XVII.] APRIL, 1863. [NO. 4.

### Original Essays and Communications.

### METALS AND ALLOYS.

BY DR. B. WOOD.

(Continued from page 75, Vol. 17.)

#### LEAD.

Hardness 1. Flex. 5. Mall. 5. Fusibility 6.

Lead freshly cut or cast presents a dark bluish-gray surface, which presently turns black owing to superficial oxidation. It is very flexible, perfectly malleable, and non-elastic. Hammering does not materially, if any, increase its specific gravity or hardness. Its specific gravity is said by some to be lessened by hammering. Prof. Graham gives its density as 11.445 which, he says, is not increased by hammering. According to the *New American Cyclopædia*, "its specific gravity when condensed by rolling is 11.44, otherwise 11.35." Its tenacity is less than that of any other ductile metal, (Graham). It has little taste, but a peculiar odor which is increased by friction. (Some authors say it has a perceptible taste, and others that it has no taste, but all agree that it has a perceptible smell when rubbed.)

Its softness is an indication of its purity, as admixtures with other metals harden it. Arsenic, however, with which it is alloyed for the purpose of shot-making, it is said, "renders the hard, brittle qualities of lead, which are contaminated by antimony and iron, softer and more ductile, and of the proper consistency to become globular." (New Am. Cy.)

Lead tarnishes quickly in the atmosphere, but the film of oxide thus formed protects the metal beneath from further oxidation. It is slowly acted on by rain water, but the salts held in spring and well water form with it an insoluble crust (generally carbonate) on the surface, which fortifies it against continued action. Most acids, even of the weaker kinds, have some action on it. Sulphuric acid attacks it only when concentrated and boiling. It dissolves, although quite slowly, in hydrochloric acid when the acid is cold as well as when hot, the testimony of some authorities to the contrary notwithstanding. Pure nitric acid dissolves it readily, but the presence of sulphuric or of hydrochloric, acid impedes this action. These acids precipitate it from the nitric solution in the form of sulphate, or of chloride, the former insoluble, the latter soluble in water. Iron and zinc precipitate it in the metallic state.

The melting point of lead is near 600° Fahr.; it is given by different experimenters as 590°, 594°, 600° and 612°, the lower figures being perhaps the more nearly exact. When melted, exposed to the air, the surface oxidizes, with rapidity proportioned to the heat, and by removing the film as it forms, the whole is soon converted into an impure protoxide, or protoxide intermixed with metallic particles, called *massicot*, which upon fusion at a red heat, whereby it is more completely oxidized, forms *litharge*. According to most authors, lead volatilizes at a white heat, but Prof. Brande says if air be carefully excluded it does not appear to be volatile at a white heat.

Lead occurs in the greatest state of purity, as well as in the greatest abundance, in the United States, being obtained from the native sulphuret, galena, a brittle mineral possessing a foliated fracture and metallic lustre, from which it is reduced by heat, with charcoal and flux. As produced from the smelting furnaces of England it always contains a little silver, and commonly zinc, antimony, copper, arsenic, one or more, or all. American lead is softer and purer.

Lead may be purified from antimony by smelting with four parts of carbonate of soda; and zinc may be entirely evaporated from it by heat, (Overman.) It is refined, in the dry way, from most admixtures, by exposing it to the action of heat and air in cupels made for the purpose, by which the oxidable contaminations separate as scoria, the silver is obtained in the metallic state, and the lead is converted into litharge from which it is reduced nearly pure.

Except iron and zinc, lead is the cheapest of metals, ranging, in the condition, as sold in commerce, from six to ten cents a pound, and of chemical purity, from six to ten cents an ounce.

The general properties of lead as an alloy with the metals before treated of have been mentioned. With cadmium and tin it forms tenacious and malleable alloys; with bismuth, and more especially with antimony, brittle ones. It renders other metals brittle if used in any considerable proportion. Tin solder consists of two parts of tin and one part of lead, is the most fusible combination of these two metals and melts sufficiently below the melting point of tin for the purpose. Plumbers' solder consists of one part of tin to from two to three parts of lead, which is sufficiently fusible for soldering lead. Lead and zinc have no mutual affinity, and do not combine chemically; their mixture for certain purposes in the arts is effected, mechanically, only by particular management. Nor do other metals appear to afford a medium of combination between the two. A mixture of several metals, as lead and zinc with tin, bismuth and antimony, or with bismuth, tin and cadmium, when stirred until congealed appears to unite more uniformly and to result in a homogeneous mass, due probably to the additional metals combining with the zinc and lead separately and forming two classes of alloys, which, by mixing, become mechanically blended.

But if left at rest to congeal, the mass separates into two portions (or more), the upper consisting of zinc chiefly if not wholly, and the lower of lead combined with the other metals; and even this lower portion is not homogeneous, the lowest part of it being softer and darker than the rest, consisting mostly of lead or of lead and bismuth. But not examining the different strata minutely, I can not hazzard any thing positive, although, thus far, it would seem that lead and zinc not only will not combine with each other directly or indirectly, but that their repulsion is so great as to prevent the perfect union of other metals with them. When melted together without other admixture, on the zinc's solidifying the lead may be drained off uncontaminated by it.

Lead unites with the hard metals, copper, silver, gold, and forms brittle alloys. It combines perfectly with platinum without the use of any flux, resulting in a brilliant button which is rather soft and quite brittle. With nickel it has little affinity, producing a sort of leaden colored slag, which does not melt but burns to a calx.

Lead, in consequence of its extensive employment in the arts, and especially the universal use of it for water-pipes and cisterns, wherein it is liable to undergo solution and to impregnate the water, in connection with the effects of its salts upon the system, has been a prolific theme with medical writers. An instance is cited where the health of a whole community was deranged by the constant use of water containing one-ninth of a grain of lead to the gallon; and another, in which several members of a household were affected after five to seven month's use of water containing one grain of lead to the gallon. Dr. John Smith, of Aberdeen, gives the "limit of manifestly deleterious action," as between one-tenth and one-twentieth of a grain to the gallon.

"Lead," says Prof. Dickson, "is a very destructive agent, when taken into the system in certain modes. Those who are engaged in making the preparations employed largely in the arts, are almost sure to suffer. Painters are so liable to these evils that rachialgia or painter's colic has thus obtained a title. Many of our beverages contain, it is said, injurious quantities of lead; cider, gin, soda water, and even fountain water, carried through lead tubes, are accused of producing disease. Nay, the exhalations of newly painted rooms, and from the clothes of those painters, have been charged with the same effects. I can not help believing that, with some truth, there is a great deal of exaggeration in these statements. * * * * * I will here add, by way of caution, that there is scarcely a salt prepared in the chemical laboratories from the great treasury of mineral and vegetable nature, that may not, in given quantity, exert a dangerous power over the body." (*Dickson's Elements of Medicine*, 1855.)

It would be interesting to examine the toxicological effects of metals, especially those used in operative dentistry, but this is not within the purport of these papers, and it would, by itself, afford an ample theme for separate discussion. But I can not forbear quoting the following information on this subject from the latest authority at hand:

"When taken into the stomach, lead is inert so long as it retains its metallic form; it begins to produce its effects only when it is oxidized. In this way bullets have been swallowed and have passed with impunity through the digestive canal. The preparations of lead vary greatly in their intensity of action, though their effects as poisons are similar; the semi-vitrified oxide (litharge), the carbonate (white lead), and the diacetate (Goulard's extract) are the most active. The sulphate, from its great insolubility in the digestive fluids, is almost if not quite inert. From the extensive use of lead in various manufactures, a great many persons are necessarily subjected to its influence. Cases of lead poisoning are common among painters, plumbers, the manufacturers of glazed cards, and those employed in the glazing of earthen ware, and in the bleaching of brussel's lace, which is beaten with white lead to whiten the fibre; plumbers who work chiefly in metallic lead suffer very much less than other artizans who employ its oxides and salts." (*New American Cyclopœdia*, 1860.) By artizans who work with it, it is inhaled in fine dust into the lungs, or into the mouth and swallowed; it is often imbibed as an adulteration in liquors. It is commonly taken into the system in small and repeated doses; occasionally in a single poisonous dose, by accident or purposely: "In this way from half an ounce to one and a half ounces of sugar of lead have been repeatedly swallowed. The symptoms have commonly been metallic taste in the mouth, burning pain in the stomach, nausea, and vomiting; to these succeeds pain in the abdomen. Sometimes the patient is purged; often there is obstinate constipation. These symptoms may subside in a day or two, or may last for ten or twelve days, combined with feeble circulation, numbness and prostration." (*Ibid.*)

Medicinally lead acts as a sedative and astringent. "It is used internally for the purpose of reducing vascular action, and restraining inordinate discharges; and externally as an abater of inflammation." (*U. S. Dispensatory.*) The carbonate, and the oxides are used externally only. The acetate (Sugar of lead) is the principle form administered internally. Of this Prof. Bache says: "Acetate of lead in medicinal doses is a powerful astringent and sedative; in larger ones, an irritant poison. The danger, however, from over-doses of sugar of lead is not so great as is generally supposed. It has sometimes been given in pretty large doses in regular practice, without any bad effects, and cases are on record where a quarter of an ounce have been swallowed without proving fatal. * * * * The principle diseases in which it has been exhibited are hemorrhages, particularly from the lungs, intestines and uterus. * * * * It has been used with advantage in certain forms of dysentery and diarrhœa, and has been recommended in particular stages of cholera infantum. * * * * It sometimes proves a valuable remedy in checking vomiting. * * * * In mercurial salivation, M. Brachet, of Lyons, found sugar of lead very efficacious, administered in grain pills night and morning. * * * * The dose of sugar of lead is from one to three grains, in the form of pill, repeated every two or three hours." (*U. S. Dispensatory.*)

The uses of lead in the arts are two various and extended to admit of enumeration here—in assaying by cupellation, in the manufacture of glass and pottery, in the making of paints, in the fabrication of alloys and metallic wares for various purposes, etc.

For dental purposes lead, undoubtedly, has been used more extensively than any other metal. Even in operative dentistry, to include the earlier operations, it has been employed more than any other. In early periods it was the chief resort for filling teeth, gold, although also resorted to, being used to only a very limited extent. John Hunter mentions only gold and lead as plugging metals, and down to about 1830 lead appears to have had no prominent rival except gold. Although about this time other metals were brought forward, still, according to certain authors who wrote at the time, it was more generally employed for filling teeth than any other material within the resources of the profession. It then partly gave place to tin and amalgam, but soon came in renewed demand for this purpose in Darcet's alloy, of which it constituted from one fourth to one third part, and which, as before mentioned, was for some years in general favor. At present, lead uncombined with other metals, is employed in France and other parts of Europe for filling teeth, to what extent we are not advised, and it is in this country not unfrequently resorted to as an anodyne or temporary stopping. It also enters as an ingredient in the "plastic metallic filling," or new fusible alloy as adapted to the purpose of filling teeth, and of which it constitutes from one-fifth to one-tenth part. In the non-plastic form of this alloy it constitutes about one-fourth, or something less.

In mechanical dentistry, lead, either uncombined, or as an alloy, is one of our chief reliances for making moulds and dies. When unalloyed it possesses a remarkable characteristic which admirably fits it for taking moulds by the dipping process. Although it shrinks greatly on congealing, it does not, like other metals, fall away from the cast, but hugs up to it, the shrinkage taking place a little beyond; so that it really remedies its own defect in this particular.

Having occupied thus much with this metal, we defer what we have to say of zinc till the next number.

ALBANY, N. Y., March, 1863.

NOTE.—*Errata.*—Page 535, Vol. 16, six lines from top, place a period after the word "impure," and let the next commence a paragraph.

For the bottom line, same page, read, "alloys formed with either of these metals are much harder than their constituents."

Page 536, six lines from top, for "compound," read "*compounds.*"

Page 24, Vol. 17, seven lines from top, for "colored," read "*closed.*"

" 25, " " twenty-four lines from top, for "discovery," read "*discoverer.*"

Page 26, Vol. 17, two lines from bottom, for "mixturs," read "*mixture.*"

" 27, " " nine lines from top, for "other," read "*either.*"

" 28, " " " " " " for "its," read "*it.*"

" 70, " " Under the caption, for "536, Vol. 16," read "30, *Vol.* 17."

" 72, " " nine lines from top, for "pacified," read "*purified.*"

" 72, " " thirteen lines from top, for "nitric," read "*nitre.*"

" 72, " " seventeen lines from top, for "hurried," read "*humid.*"

" 72, " " twenty-five lines from top, for "alloy," read "*alloys.*"

" 74, " " seven lines from top, for "serullees," read "*serullas.*"

" 75, " " two lines from top, for "dyes," read "*dies.*"

Also a few orthographical misprints immaterial to the sense.

